# The Impact of a Mobile Diabetes Health Intervention on Diabetes Distress and Depression Among Adults: Secondary Analysis of a Cluster Randomized Controlled Trial

**DOI:** 10.2196/mhealth.8910

**Published:** 2017-12-07

**Authors:** Charlene C Quinn, Krystal K Swasey, J Christopher F Crabbe, Michelle D Shardell, Michael L Terrin, Erik A Barr, Ann L Gruber-Baldini

**Affiliations:** ^1^ Department of Epidemiology and Public Health School of Medicine University of Maryland Baltimore, MD United States; ^2^ School of Social Work Fordham University Baltimore, NY United States

**Keywords:** diabetes distress, mobile health, depression, diabetes, Diabetes Distress Scale, Patient Health Questionnaire, women, emotional well-being

## Abstract

**Background:**

Diabetes is a complex, demanding disease that requires the constant attention of patients. The burden of self-management, including different medication regimens, routine self-care activities, and provider visits, has an impact on patients’ emotional well-being. Diabetes distress and depression are two important components of emotional well-being that may negatively affect diabetes outcomes.

**Objective:**

The aim was to determine the impact of the 1-year Mobile Diabetes Intervention Study cluster randomized clinical trial on emotional well-being measured by diabetes distress and depression among adults with type 2 diabetes (T2D).

**Methods:**

A total of 163 adults with not-well-managed T2D were enrolled from community primary care practices. Primary care practices were cluster randomized into either a usual care control group or intervention group. Intervention participants were given a mobile phone with coaching software including a Web portal to communicate with providers. A priori established secondary outcomes included distress measured by the Diabetes Distress Scale (DDS), with subscales measuring emotional burden, interpersonal distress, physician-related distress, and regimen-related distress, as well as depression measured by the Patient Health Questionnaire (PHQ-9). Linear mixed models were used to calculate the effect of the intervention on diabetes distress levels over time, both overall and separately by sex, and to determine if the intervention affected distress or depression. The impact of total DDS on changes in HbA_1c_ was also studied.

**Results:**

There were no significant treatment group effects for DDS total (baseline: *P*=.07; differences over time: *P*=.38) or for depression (*P*=.06 over time). Significant declines in total DDS were observed over the 12-month intervention period (*P*=.01). Regimen-related distress significantly decreased for all study participants (*P*<.001), but no significant change over time was observed for emotional burden (*P*=.83), interpersonal distress (*P*=.64), or physician-related distress (*P*=.73). Women in both the usual care and intervention groups were more likely to have higher overall DDS, emotional burden, physician-related distress, and regimen-related distress, but not interpersonal distress. Women also reported higher baseline depression compared to men (*P*=.006). Overall, depression decreased over the treatment period (*P*=.007), but remained unaffected by group assignment (*P*=.06) or by sex (*P*=.97). Diabetes distress had no effect on the change in HbA_1c_ (*P*=.91) over the treatment period.

**Conclusions:**

Although we found no definitive overall or sex-specific effect of the intervention on diabetes distress or depression, this study makes an important contribution to the understanding of mobile health interventions and the impact on emotional health. Our study verified previous work that although diabetes distress and depression are highly correlated, these measures are not evaluating the same construct. Design of future mobile technology provides an opportunity to personalize, contextualize, and intervene in the emotional well-being of persons with diabetes.

**Trial Registration:**

Clinicaltrials.gov NCT01107015; https://clinicaltrials.gov/ct2/show/NCT01107015 (Archived by WebCite at http://www.webcitation.org/6vVgRCLAF)

## Introduction

Diabetes affects an estimated 30.3 million people in the United States [1]. Diabetes prevalence continues to increase across all age, minority, and income groups [2,3]. It is projected that one in three US adults will be diagnosed with diabetes by 2050 [4]. Type 2 diabetes (T2D) comprises 90% of diabetes and is caused by modifiable lifestyle factors, genetics, and aging. As a result of long exposure to the physiological consequences of T2D—unmanaged blood glucose, blood pressure, and cholesterol—individuals may experience complications. These complications may affect individuals’ physical and emotional well-being, making diabetes a challenging and demanding disease to manage.

Previous studies have shown that the first line of defense to delay or manage diabetes complications is through self-management practices [5,6]. In addition to multiple daily self-care activities, patients experience multiple medication regimens, high out-of-pocket health expenses, complication-specific treatments, and interactions with five or more health providers that can add up to as much as two hours each day spent managing their diabetes [7,8]. When considered together, the burden of these chronic disease factors may have a long-term impact on psychological functioning or emotional well-being [9]. Two components of emotional well-being include diabetes distress and depression. Diabetes distress is conceptually different from diabetes-related depression, and evaluation and treatment of diabetes distress has clinical utility because moderate to high distress is related to poor diabetes outcomes [10].

### Diabetes Distress

Diabetes distress includes emotional responses to the diabetes diagnosis, risk of complications, self-management demands, unresponsive providers, and/or indifferent interpersonal relationships [11,12]. Feeling that family, friends, and even health care providers do not fully understand the everyday struggles of living with the chronic disease may further create an isolating experience for patients with diabetes. Complex and perhaps confusing daily diabetes self-management regimens may be overwhelming. Diabetes distress does not affect both sexes equally. In previous studies, women reportedly had a greater relative risk of experiencing diabetes distress [13,14], as well as higher odds of becoming distressed over an extended treatment period compared to men [15].

One of the most frequently used measures of diabetes distress is the Diabetes Distress Scale (DDS). This 17-item self-report questionnaire is used to gauge physician-related distress as well as problems related to diabetes self-management, self-care, and metabolic outcomes [16]. To establish clinical meaningfulness, the DDS total and its subscales were studied in relation to diabetes-specific clinical (glycated hemoglobin A_1c_ [HbA_1c_]) and behavioral (self-efficacy, diet, physical activity) variables. High DDS and increases in distress are associated with poorer outcomes: high HbA_1c_, low self-efficacy, and not choosing healthy foods [17].

### Diabetes Distress Scale and Depression

Within the United States, the high prevalence of depressive symptoms among patients with diabetes—between 18% and 35%—has been well-documented [18,19]. Depression with diabetes is associated with suboptimal disease management, inadequate glycemic control, higher functional impairment [20,21], and risk of diabetes complications [22-24]. Although there are similarities, diabetes distress is not indicative of depression—research shows that diabetes distress is related to, but distinct from, major depressive disorder [24,25]. This may explain why treatment of depression in patients with diabetes may have little effect on diabetes management [24].

Creating technological interventions to improve diabetes outcomes is not new, yet as mobile device use increases, these interventions are becoming even more widespread [26]. Several studies that incorporate digital health interventions have reported overall success in reducing depressive symptoms [27] and improving diabetes outcomes such as HbA_1c_ [28-31], self-management, and self-efficacy [31]. However, few studies have evaluated the intervention effects on DDS total or DDS subscale variables and depression. The REDEEM study compared three interventions with varying degrees of computer-assisted self-management to reduce diabetes distress and improve self-management with non-clinically depressed adults with T2D. Across all intervention conditions, REDEEM investigators observed significant reductions in DDS, emotional burden, and regimen-related distress. Self-management behaviors also improved, such as healthy eating and medication adherence, but not HbA_1c_ [32].

Diabetes and its complications affect a substantial number of Americans, and there is lack of evidence on intervention strategies that meet their emotional needs [32,33]. As technology advances, interventions may reach new audiences and promote better diabetes outcomes if presented in the convenience of a mobile platform. The primary aim of this secondary analysis was to determine the impact of a one-year mobile diabetes intervention on diabetes distress and depression among adults with T2D. We also aimed to examine the effect of diabetes distress on HbA_1c_.

## Methods

### Participants and Procedures

Details of the Mobile Diabetes Intervention Study (MDIS) have previously been reported [34-36]. Secondary analyses pertaining to emotional health reported here were established a priori in the protocol.

Eligibility criteria for this cluster randomized controlled trial (RCT) included physician diagnosis of diabetes within the 6 months prior to enrollment, HbA_1c_ level of 7.5% or higher within the previous 3 months, English speaking, and age between 18 and 64 years. Patients were deemed ineligible for participation if they were beneficiaries of Medicare or Medicaid, were uninsured, used an insulin pump, were pregnant, had actively abused alcohol or drugs within the past year, were being treated for psychosis or schizophrenia, suffered from severe, uncorrected hearing or vision impairment, or if they did not have an email address and access to the Internet [34,35]. At the time of consent, patients knew their assignments to control or intervention.

The study was conducted in primary care settings within four Maryland areas. Each patient was randomized at the physician-practice level (cluster) to either the control group (group 1: control-usual care), or one of three intervention groups: coach only, coach primary care provider (PCP) portal, and coach PCP portal with decision support. The coach-only and coach-PCP portal groups were prespecified as ancillary to the study design of the clinical trial and were not included in this analysis. Therefore, N=114 for this analysis.

### Intervention

The aim of this analysis was to determine whether a mobile phone Web-based portal intervention and messaging communication intervention had an effect on diabetes-related distress and depression. The control-usual care patients received care as usual provided by their primary care physicians. Participants in the intervention group, coach PCP portal with decision support, had access to a mobile coaching system, which collected and analyzed glucose trends over a 1-year period. The physicians of patients in this group had full access to patient data via Web portals and were given summarized reports with patient treatment recommendations every 3 months. All patients received a One Touch Ultra 2TM (LifeScan, Milpitas, CA, USA) glucose meter and blood glucose testing supplies for a year. Physicians of patients in the control group were instructed to provide care as usual.

Patients in the coach PCP portal with decision support group received a multitier coaching system aimed at gaining control over the disease. Patients received one of two mobile phone models, a 1-year unlimited data and service plan and coaching software on their mobile phones to communicate their diabetes-specific information. For example, patients could enter their blood glucose levels, carbohydrates consumed, diabetes medications taken, and any comments about their diabetes self-care, all recorded in real time in a Web-based logbook. Participants received automated self-management messages specifically tailored to the values they entered, longitudinal data trends, and their physicians’ individualized medication instructions. Patients could also receive communications from their study certified diabetes educator or endocrinologist by communicating through secure messages on a patient-specific Web portal. Messages included automatic information responding to patient-reported values (ie, blood glucose) and reminders to use educational materials on the patient Web portal directed at diabetes self-management behaviors. Messages and interactions with diabetes educators included managing daily regimens, worries about complications, and anxiety about poor disease management, but did not target specific concepts in the DDS or DDS subscales.

### Study Oversight

The Institutional Review Board of the University of Maryland, Baltimore, MD, approved this study. After study enrollment was closed, errors in consent form completion were found on audit. To assure that we obtained the appropriate signatures, the Institutional Review Board asked us to repeat our consent procedures, which we did for 163 patient participants and all 39 physician participants. Patients excluded due to not receiving reconsent did not significantly differ (*P*>.10) at baseline from included patients in age, gender, or baseline HbA_1c_. A Data and Safety Monitoring Board was appointed to review the study procedures and adverse events.

### Measures

Demographic characteristics including sex, age, and race were patient self-reported and confirmed through medical chart review. Education and smoking status were self-reported by patients during study interviews. Trained staff blinded to group assignment used the Bayer DCA 2000 to measure HbA_1c_. If HbA_1c_ levels were not obtained within 14 days of the conclusion of the 12-month study, reminders were sent to both physicians and patients to complete the test. Measures of systolic blood pressure, diastolic blood pressure, low-density lipoprotein cholesterol, high-density lipoprotein cholesterol, triglycerides, total cholesterol, and medications have previously been reported [35,36]. Study data for primary and secondary outcomes were collected by research staff separately from data transmitted through the mobile device.

Diabetes distress and depression were measured by the 17-item DDS and the nine-item Patient Health Questionnaire (PHQ-9). The DDS asks respondents to rate the degree to which diabetes situations caused distress or bothered the person during the previous month [25]. Four subscales have been identified as distinct areas of potential diabetes distress: emotional burden assesses the extent patients with diabetes feel overwhelmed by their disease, physician-related distress addresses the availability and open communication with health care providers, interpersonal distress assesses feelings pertaining to the support of family and friends, and regimen-related distress evaluates patients’ preparedness to adhere to meal plans, monitoring blood glucose levels, and taking medications [17]. The DDS, including the four subscales, was measured at baseline and at the end of the intervention (12 months). Internal consistency has been assessed by the alpha coefficient (0.93 for the total scale and 0.88 to 0.90 for the four subscales) [17]. The DDS asks individuals to rate each item on a Likert-like scale from 1 (not a problem) to 6 (a very serious problem) [25]. The total DDS score is based on averaging responses across items; therefore, the total DDS ranges from 1 to 6 [25]. For this analysis, we summarized total DDS scores in three categories: little or no diabetes distress (DDS <2.0), moderate diabetes distress (DDS=2.0-2.9), and high diabetes distress (DDS ≥3), which were previously documented to have clinical meaning [37]. The DDS subscale measures, emotional burden, physician-related distress, regimen-related distress, and interpersonal distress were also analyzed. To score the subscales, the sum of patient responses for the subscale items were divided by the number of items in that subscale. Emotional burden included five items (1, 3, 8, 11, and 14), physician-related distress included four items (2, 4, 9, and 15), regimen-related distress included five items (5, 6, 10, 12, and 16), and interpersonal distress included three items (7, 13, and 17).

Depression was assessed by the PHQ-9 at baseline and at 12-month study end. The PHQ-9, tested in primary care, has demonstrated clinical relevance to *Diagnostic and Statistical Manual of Mental Disorders* (Fourth Edition) depression criteria and is used as a research diagnosis of depression. The PHQ-9 scores range from 0 to 27, with scores indicating minimal depression (0-4), mild depression (5-9), moderate depression (10-14), moderately severe depression (15-19), and severe depression (≥20) [38].

### Statistical Analysis

The main comparison for this analysis stated a priori was between the control group and the intervention group (coach PCP portal with decision support). Means and standard deviations for continuous variables, and frequencies and proportions for categorical variables were analyzed. Means of total DDS scores and the four subscales (emotional burden, physician-related distress, regimen-related distress, and interpersonal distress) were calculated by treatment group and sex. Linear mixed models were used to examine the effects of the intervention over time both overall and separately by sex on levels of diabetes distress while accounting for practice level (cluster) randomization. Linear mixed models were also used to determine whether the mobile health intervention impacted changes in total DDS, DDS subscale measures, and depression, and whether the effects differed by sex. Correlations for DDS, DDS subscales, and PHQ-9 were computed to examine if diabetes distress and depression were measuring the same construct in this population. SAS version 9.2 (SAS Institute Inc, Cary, NC, USA) was used to perform all statistical analyses. A *P*<.05 was considered statistically significant.

## Results

Baseline characteristics of study patients are shown in Table 1. Mean age was 52.6 (SD 8.2) years and the duration of diabetes diagnosis was mean 8.5 (SD 6.1) years. In all, 56.1% (64/114) were white and 71.9% (82/114) had at least some college education. Of the 114 participants, 37 (32.5%) had mild diabetes distress and 44 (38.6%) had moderate diabetes distress at baseline (Table 1).

**Figure 1:**
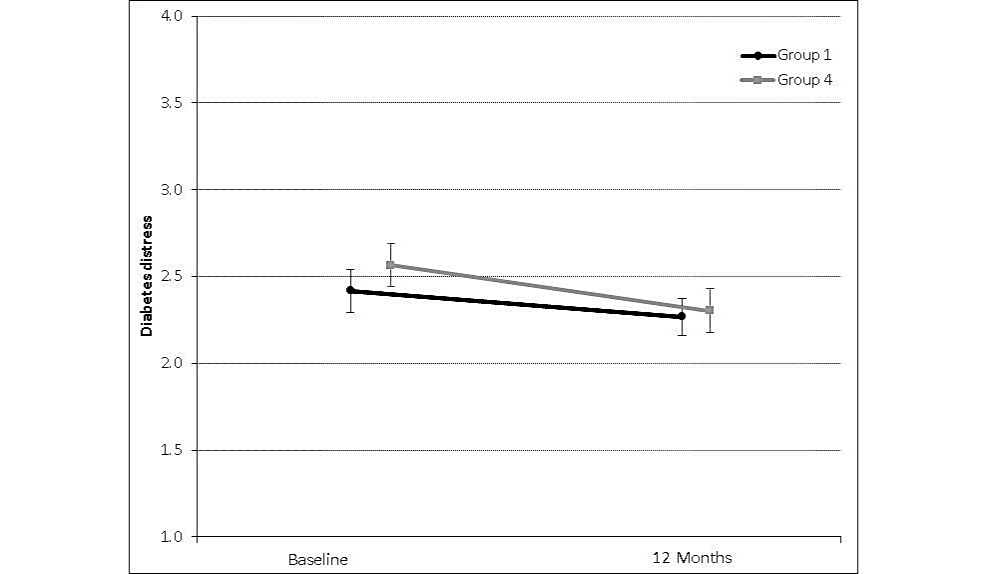
Mean and standard deviation for the change in total Diabetes Distress Scale scores from baseline to 12 months for control (n=46-56) and intervention (n=57-58) groups. Whiskers represent standard deviation.

Significant declines in total DDS scores were observed over the 12-month intervention period (*P*=.01) (Table 2). However, group assignment did not significantly affect total DDS scores (*P*=.79) (Table 2 and Figure 1). Furthermore, differences in total DDS score changes between the usual care and coach PCP portal with decision support groups were not significant (*P*=.38) (Table 2).

### Diabetes Distress Scale Subscales

**Figure 2:**
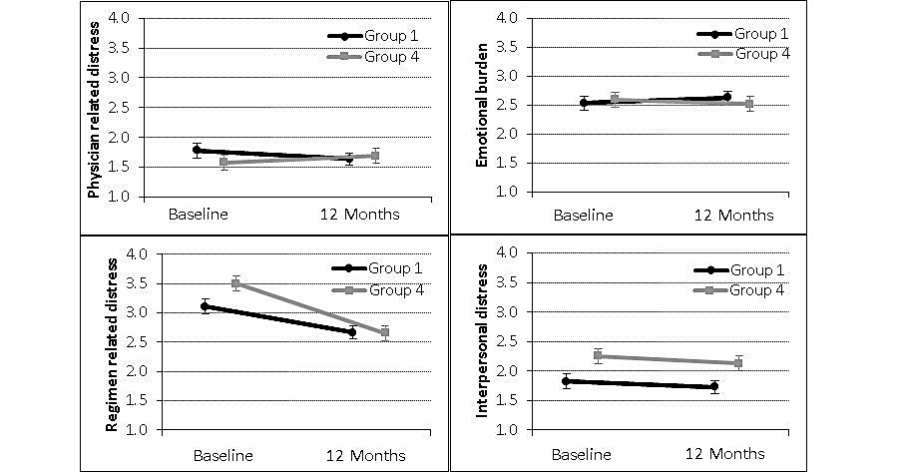
Mean and standard deviation for the change in Diabetes Distress Scale subscale scores from baseline to 12 months for control (n=46-56) and intervention (n=57-58) groups. Whiskers represent standard deviation.

Regimen-related distress showed the highest mean of all subscales at baseline (mean 3.3, SD 1.3), corresponding to high diabetes distress (Table 2). Among all study participants, regimen-related distress significantly changed over the 12-month treatment period (*P*<.001) (Table 2 and Figure 2). Emotional burden (*P*=.83), physician-related distress (*P*=.73), and interpersonal distress (*P*=.64) did not significantly change over 12 months. The DDS subscales did not significantly differ at baseline and changes in subscale scores over time did not significantly differ (Table 2).

### Diabetes Distress Scale and Sex

Baseline DDS total scores were significantly different between males and females, regardless of group assignment (*P*=.002). Sex differences at baseline were observed for all but one DDS subscale scores including emotional burden (*P*=.04), regimen-related distress (*P*=.01), physician-related distress (*P*=.009), but not interpersonal distress (*P*=.08). However, the effect of the intervention on total diabetes distress and the subscale scores did not significantly differ by sex (Table 3). Males had lower total DDS, emotional burden, regimen-related distress, and physician-related distress than females.

At baseline, women had higher depression than men did (*P*=.006). There was no significant change over time by sex (*P*=.97). Linear mixed models determined that PHQ-9 scores significantly declined over the 12-month study period (*P*=.007), but did not significantly differ by treatment group (*P*=.06). Correlational analyses found that higher baseline diabetes distress (DSS total) was significantly associated with higher depression (*r*=.57, *P*<.001). Subscale correlations with PHQ-9 were moderate: emotional burden (*r*=.53, *P*<.001), interpersonal distress (*r*=.46, *P*<.001), regimen-related distress (*r*=.44, *P*<.001), and physician-related distress (*r*=.21, *P*=.009).

We previously reported that HbA_1c_ levels of study participants significantly decreased over the 12-month treatment period [34]. In this analysis, linear mixed models determined that baseline DDS scores were not significantly associated with baseline HbA_1c_ (*P*=.45) nor were changes in DDS associated with HbA_1c_ change over time (*P*=.91).

**Table 1:** Baseline characteristics of patients and primary and secondary outcomes.

Baseline characteristics		Both groups	Control-usual care			Coach PCP^a^ portal with decision support			
		(n=114)	All (n=56)	Male (n=28)	Female (n=28)		All (n=58)	Male (n=30)	Female (n=28)
**HbA_1c_^b^ (%), mean (SD^c^)**		9.6 (2.0)	9.2 (1.7)	9.2 (1.7)	9.1 (1.7)		9.9 (2.1)	9.8 (2.3)	10.1 (2.0)
	7.5%-8.9%, n (%)	61 (53.5)	35 (62.5)	17 (60.7)	18 (64.3)		26 (44.8)	15 (50.0)	11 (39.3)
	≥9%, n (%)	53 (46.5)	21 (37.5)	11 (39.3)	10 (35.7)		32 (55.2)	15 (50.0)	17 (60.7)
Age (years), mean (SD)		52.6 (8.2)	53.2 (8.4)	51.9 (8.4)	54.6 (8.2)		51.9 (8.1)	54.3 (6.7)	49.4 (8.7)
**Race, n (%)**									
	Nonwhite	50 (43.9)	30 (53.6)	14 (50.0)	16 (57.1)		20 (34.5)	7 (23.3)	13 (46.4)
	White	64 (56.1)	26 (46.4)	14 (50.0)	12 (42.9)		38 (65.5)	23 (76.7)	15 (53.6)
**Duration of diabetes**									
	Diagnosis (years), mean (SD)	8.5 (6.1)	9.0 (7.0)	7.3 (4.4)	10.7 (8.6)		8.1 (5.3)	7.5 (4.9)	8.7 (5.7)
**Smoking status, n (%)**									
	Current smoker	19 (16.7)	11 (19.6)	5 (17.9)	6 (21.4)		8 (13.8)	5 (16.1)	3 (9.7)
	Not current smoker	95 (83.3)	45 (80.4)	23 (82.1)	22 (78.6)		50 (86.2)	26 (83.9)	28 (90.3)
**Education, n (%)**									
	High school/trade school or less	32 (28.1)	14 (25.0)	5 (17.9)	9 (32.1)		18 (31.0)	12 (40.0)	6 (21.4)
	Some college or associates	41 (36.0)	20 (35.7)	10 (35.7)	10 (35.7)		21 (36.2)	8 (26.7)	13 (46.4)
	Bachelors degree or higher	41 (36.0)	22 (39.3)	13 (46.4)	9 (32.1)		19 (32.8)	10 (33.3)	9 (32.1)
Body mass index (kg/m²), mean (SD)		35.3 (6.8)	34.3 (6.3)	33.8 (6.3)	34.8 (6.4)		36.2 (7.1)	33.2 (4.5)	39.4 (8.0)
**Comorbidities, n (%)**									
	Hypertension	69 (60.5)	29 (51.8)	14 (50.0)	15 (53.6)		40 (69.0)	21 (70.0)	19 (67.9)
	Hypercholesterolemia	66 (57.9)	34 (60.7)	17 (60.7)	17 (60.7)		32 (55.2)	20 (66.7)	12 (42.9)
	Coronary artery disease	10 (8.8)	5 (8.9)	4 (14.3)	1 (3.6)		5 (8.6)	3 (10.0)	2 (7.1)
	Microvascular complications (any)	14 (12.3)	8 (14.3)	4 (14.3)	4 (14.3)		6 (10.3)	3 (10.0)	3 (10.7)
**Depression (PHQ-9^d^)**									
	Score (0-27), mean (SD)	5.1 (5.5)	4.7 (5.6)	3.2 (4.2)	6.2 (6.5)		5.5 (5.4)	4.8 (5.4)	6.3 (5.5)
	Minimal to mild (0-9), n (%)	90 (78.9)	45 (80.4)	26 (92.9)	19 (67.9)		45 (77.6)	25 (83.3)	20 (71.4)
	Moderate to severe (10-27), n (%)	24 (21.1)	11 (19.6)	2 (7.1)	9 (32.1)		13 (22.4)	5 (16.7)	8 (28.6)
**Diabetes Distress Scale, n (%)**									
	Little or no distress (DDS <2.0)	37 (32.5)	20 (35.7)	11 (39.3)	9 (32.1)		17 (29.3)	13 (43.3)	4 (14.3)
	Moderate distress (DDS=2.0-2.9)	44 (38.6)	21 (37.5)	10 (35.7)	11 (39.3)		23 (39.7)	13 (43.3)	10 (35.7)
	High distress (DDS ≥3)	33 (28.9)	15 (26.8)	7 (25.0)	8 (28.6)		18 (31.0)	4 (13.3)	14 (50.0)

^a^PCP: primary care provider.

^b^HbA_1c_: glycated hemoglobin A_1c_.

^c^SD: standard deviation.

^d^PHQ-9: Patient Health Questionnaire.

**Table 2:** Diabetes Distress Scale (DDS)^a^ and subscale scores.

Study measures			Both groups (n=114)				Usual care (n=56)				Coach PCP^b^ portal with decision support (n=58)				*P*^c,d^	
			n	Mean (SD^e^)		*P*^f^		n		Mean (SD)		n		Mean (SD)		
**DDS**																
	Baseline	114		2.5 (0.9)			56		2.4 (0.9)		58		2.6 (0.9)		.79^c^	
	12 month	103		2.3 (0.8)			46		2.3 (0.9)		57		2.3 (0.8)			
	Change	103		–0.2 (0.8)	.01		46		–0.1		57		–0.3		.37^d^	
**Emotional burden**																
	Baseline	114		2.6 (1.3)			56		2.6 (1.3)		58		2.6 (1.3)		.75^c^	
	12 month	104		2.6 (1.3)			46		2.6 (1.3)		58		2.5 (1.2)			
	Change	104		0 (1.2)	.83		46		0.1		58		–0.1		.48^d^	
**Interpersonal distress**																
	Baseline	114		2.1 (1.3)			56		1.9 (1.1)		58		2.3 (1.4)		.24^c^	
	12 month	103		2.0 (1.2)			46		1.8 (1.1)		57		2.2 (1.2)			
	Change	103		0 (1.2)	.64		46		0.1		57		–0.1		.65^d^	
**Physician-related distress**																
	Baseline	114		1.7 (1.0)			56		1.8 (1.0)		58		1.6 (0.9)		.86^c^	
	12 month	104		1.7 (1.0)			46		1.7 (1.0)		58		1.7 (1.0)			
	Change	104		0 (1.0)	.73		46		–0.2		58		0.1		.12^d^	
**Regimen-related distress**																
	Baseline	114		3.3 (1.3)			56		3.1 (1.3)		58		3.5 (1.2)		.65^c^	
	12 month	104		2.7 (1.1)			46		2.7 (1.1)		58		2.7 (1.0)			
	Change	104		–0.6 (1.4)	<.001		46		–0.4		58		–0.8		.16^d^	

^a^The three categories of DDS scores are little or no diabetes distress (DDS <2.0), moderate diabetes distress (DDS=2.0-2.9), and high diabetes distress (DDS ≥3) [36].

^b^PCP: primary care provider.

^c^Group effect on diabetes distress, regardless of gender.

^d^Group by time effect on diabetes distress, regardless of gender.

^e^SD: standard deviation.

^f^Time effect on diabetes distress scores in all groups.

**Table 3:** Diabetes Distress Scale (DDS)^a^ and subscale scores by sex.

Study measures		Both groups (n=114)			Usual care					Coach PCP^b^ portal with decision support						*P*^c^	
		n	Mean (SD^d^)	*P*^e^	Male (n=28)		Female (n=28)			Male (n=30)			Female (n=28)				
					n	Mean (SD)		n	Mean (SD)		n	Mean (SD)		n	Mean (SD)		
**DDS**																	
	Baseline	114	2.5 (0.9)		28	2.4 (1.0)		28	2.5 (0.9)		30	2.2 (0.7)		28	3.0 (0.9)		
	12 month	103	2.3 (0.8)		23	2.2 (0.8)		23	2.4 (0.9)		29	2.0 (0.7)		28	2.6 (0.7)		
	Change	103	–0.2 (0.8)	.01	23	–0.1 (0.8)		23	–0.1 (0.7)		29	–0.1 (0.7)		28	–0.4 (0.8)		.23
**Emotional burden**																	
	Baseline	114	2.6 (1.3)		28	2.3 (1.2)		28	2.8 (1.4)		30	2.3 (1.2)		28	3.0 (1.4)		
	12 month	104	2.6 (1.3)		23	2.4 (1.0)		23	2.9 (1.6)		30	2.4 (1.2)		28	2.7 (1.3)		
	Change	104	0 (1.2)	.83	23	0.1 (1.0)		23	0.2 (1.3)		30	0.1 (1.1)		28	–0.2 (1.3)		.41
**Interpersonal distress**																	
	Baseline	114	2.1 (1.3)		28	2.0 (1.0)		28	1.8 (1.1)		30	1.8 (0.9)		28	2.8 (1.6)		
	12 month	103	2.0 (1.2)		23	1.7 (0.9)		23	1.9 (1.3)		29	1.9 (1.0)		28	2.5 (1.4)		
	Change	103	0 (1.2)	.64	23	0.0 (0.8)		23	0.2 (1.3)		29	0.0 (1.0)		28	–0.3 (1.6)		.18
**Physician-related distress**																	
	Baseline	114	1.7 (1.0)		28	1.8 (1.1)		28	1.8 (1.0)		30	1.2 (0.4)		28	2.0 (1.1)		
	12 month	104	1.7 (1.0)		23	1.7 (0.9)		23	1.6 (1.1)		30	1.3 (0.6)		28	2.1 (1.1)		
	Change	104	0 (1.0)	.73	23	–0.2 (0.8)		23	–0.2 (0.9)		30	0.1 (0.6)		28	0.2 (1.4)		.77
**Regimen-related distress**																	
	Baseline	114	3.3 (1.3)		28	3.1 (1.3)		28	3.1 (1.3)		30	2.9 (1.1)		28	4.0 (1.1)		
	12 month	104	2.7 (1.1)		23	2.6 (1.1)		23	2.7 (1.0)		30	2.3 (0.9)		28	3.0 (1.0)		
	Change	104	–0.6 (1.4)	<.001	23	–0.4 (1.6)		23	–0.3 (1.1)		30	–0.6 (1.3)		28	–1.0 (1.5)		.38

^a^The three categories of DDS scores are little or no diabetes distress (DDS <2.0), moderate diabetes distress (DDS=2.0-2.9), and high diabetes distress (DDS ≥3) [36].

^b^PCP: primary care provider.

^c^Treatment by gender effect on diabetes distress.

^d^SD: standard deviation.

^e^Time effect on diabetes distress scores in all groups.

## Discussion

### Principal Results

In the MDIS, we hypothesized that the intervention would reduce diabetes distress, since self-management of diabetes during periods between health care provider visits can be challenging for patients. Lack of interactive communication with health care providers may leave patients unmotivated to maintain their diabetes regimen. Unlike the REDEEM study [32], we found no overall effect of the intervention on diabetes distress or depression, nor did we find treatment differences by sex. We also observed that DDS total had no significant impact on the change in HbA_1c_ over time. These findings may be due to study participants who at baseline had overall mean low-moderate diabetes distress and overall mean low-moderate distress conditions (ie, emotional burden interpersonal distress, physician-related distress, and regimen-related distress). Another explanation may be that for individual participants who had moderate to high levels of distress, participating in the intervention added to their disease burden [7,8,39]. Despite these null results, we found that regimen-related distress, total DDS, and depression significantly decreased over the treatment period. Women were significantly more depressed and had higher baseline DDS total scores, emotional burden, regimen-related distress, and physician-related distress scores compared to men.

In a behavioral RCT, Hessler et al [40] also tested an intervention to reduce diabetes distress, but included participants with at least a moderate level of regimen-related distress. Cross-sectional, prospective model analyses within the study identified significant time-varying findings that suggested decreases in regimen-related distress were associated with improved medication adherence, physical activity, and HbA_1c_ over time [40]. The authors suggest that linkages found among regimen-related distress and glycemic control may be explained by biological (hormonal), behavioral (nonadherence), and affective (burden of diabetes management) factors.

Spring et al [41] showed that the effect of mobile feedback on a targeted behavior (eating more fruits and vegetables) had a reciprocal effect on an untargeted behavior (physical activity). In the MDIS, we did not target the intervention on a single behavior to reduce distress. It may be that the impact of change in one diabetes distress subscale on another over time is complex, reciprocal, and iterative. Therefore, it may be important for future mobile diabetes studies to assess multiple causal pathways of emotional well-being with differences evaluated among various patient populations [42].

The Diabetes Attitudes, Wishes and Needs (DAWN2) study was groundbreaking in addressing psychosocial issues [43] including diabetes distress, the value of team care, inclusion of family members [44], and importance of behavioral needs of patients [45]. Despite the evidence for effectiveness of diabetes self-management [46,47], DAWN2 reported low participation rates for patients and family groups, with participants reporting education and psychosocial support are seldom available [45]. Advances in mobile technology that enable us to track individual behavior linked to clinical measures within contextual factors (disease severity, comorbidities, age, resources, and distress factors) afford us the opportunity to engage patients and personalize education or emotional needs to address diabetes distress [48].

Multiple causal pathways may also explain the impact of diabetes distress on changes in depression over time. Our study verified previous work [32] that although diabetes distress (as measured by DDS) and depression are highly correlated, these measures are not evaluating the same construct (*r*=.57). Despite patients having less-than-well-managed diabetes at baseline in the MDIS, participants were not substantially depressed. Therefore, it was difficult to measure improvement in depression over time. Participants in our study were diagnosed with diabetes for a mean 8.5 (SD 6.1) years and may have developed coping strategies as suggested by Fisher et al [32]. Also in the MDIS, patients knew study group assignment at baseline; self-selection may have influenced their initial level of distress. One option for future mobile health studies would be to consider collecting mobile data on trait-like variables (mindful eating, positive affect, feeling empowered) [49-51] shown to influence distress, disease management, and health outcomes (HbA_1c_) [50]. Also, concealing treatment assignments at baseline evaluation could lead to more sensitive comparisons.

In this analysis, we also explored the role of patient sex on diabetes distress. Diabetes distress was higher among women than men at baseline, but there was no difference by sex over the treatment period. Women also experienced higher levels of regimen-related distress, physician distress, and emotional burden, but no difference from men in interpersonal distress. Previous studies of similar community populations with T2D and diabetes distress have not found differences by sex [37].

To our knowledge, this is the first cluster RCT to report a mobile health evaluation of emotional well-being and diabetes clinical outcomes. This study has several strengths, including a population with T2D treated by PCPs in which 90% of diabetes care is provided, use of validated measures of diabetes distress and depression, and a 12-month intervention study of an important clinical measure of diabetes (HbA_1c_).

### Limitations

Although this analysis had many strengths, there are limitations that should be considered when interpreting our findings. The study was not powered for this secondary analysis, and the small sample size within groups may have obscured true moderate-sized differences. The exclusion of participants who had Medicare or Medicaid limited our sample size; of those diagnosed with diabetes, 97.6% and 89.9% of individuals with these payer types have type 2 diabetes, respectively [52]. Also, we did not measure participant engagement in the intervention during the 12-month period. If patients did not enter their glucose levels, medication use, or other diabetes-specific information into their mobile devices regularly, they would receive fewer automated self-management messages and thus potentially have missed the opportunity to reduce feelings of distress or depression. Specific participant demographics, such as race, age, health literacy, or proximity of diagnosis to study treatment, may influence the utilization of a mobile health intervention [53,54]. Although participants in our study had low levels of diabetes distress and depression at baseline, it may be that the technology design, including messaging content, should address potential disease burden which impacts emotional health.

### Conclusion

Although we found no definitive overall or sex-specific effect of the intervention on diabetes distress or depression, this study does make an important contribution to the understanding of mobile health interventions and the impact on emotional health. Design of future mobile technology provides an opportunity to personalize, contextualize, and intervene in the emotional well-being of persons with diabetes.

## Abbreviations

DDS: Diabetes Distress Scale
HbA_1c_: glycated hemoglobin A_1c_
MDIS: Mobile Diabetes Intervention Study
PCP: primary care provider
PHQ-9: Patient Health Questionnaire-9 item
RCT: randomized controlled trial
T2D: type 2 diabetes
